# Analysis of blood culture practices in multicenter university-affiliated hospitals in Korea: A retrospective study focused on procedural optimization

**DOI:** 10.1371/journal.pone.0337816

**Published:** 2025-12-29

**Authors:** Sae Am Song, Sunggyun Park, Kwangsook Woo, Yu Kyung Kim, Jung-ah Kim, Sunjoo Kim

**Affiliations:** 1 Department of Laboratory Medicine, Inje University College of Medicine, Busan, Korea; 2 Departments of Laboratory Medicine, Keimyung University School of Medicine, Daegu, Korea; 3 Department of Laboratory Medicine, Dong-A University College of Medicine, Busan, Korea; 4 Department of Clinical Pathology, School of Medicine, Kyungpook National University, Daegu, Korea; 5 Department of Laboratory Medicine, Soonchunhyang University Seoul Hospital, Seoul, Korea; 6 Department of Laboratory Medicine, Gyeongsang National University College of Medicine, Jinju, Korea; University of Bologna / Romagna Local Health Authority, ITALY

## Abstract

**Background:**

Blood culture is an essential diagnostic tool for detecting bloodstream infections, particularly in patients with suspected sepsis. Routine implementation in healthcare settings necessitates adherence to standardized protocols to ensure diagnostic accuracy. We assessed blood culture practices in university-affiliated hospitals across Korea by analyzing key performance metrics and related indicators.

**Methods:**

In 2024, a standardized questionnaire was distributed to 14 university-affiliated hospitals. The survey collected data on blood culture practices during 2023, including the total number of cultures performed, the number of sets obtained per episode, the frequency of repeated cultures, the volume of blood collected per bottle, discrepancies in culture results, the types of organisms isolated, and post-test reporting practices.

**Results:**

In 2023, the median number of blood cultures requested per 1,000 hospitalized patients was 166.6 (43,699 cultures among 241,053 admissions). Two-set collections were most common (median 68.9%), followed by single-set collections (13.0%). Repeat cultures were more frequently performed following negative results (29.2%) than positive ones (5.5%). The median volume collected was 4.0 and 4.3 mL for aerobic and anaerobic bottles, respectively. Only 16.7% of Gram stain results were reported within 2 h.

**Conclusions:**

Blood culture practices, particularly regarding test ordering and the use of two or more sets per episode, appear generally appropriate. However, specific aspects—including the indications for repeat cultures, adequacy of collected blood volume, and timeliness of Gram stain reporting—require further evaluation and targeted quality improvement efforts.

## Introduction

Blood culture remains the gold standard for detecting bacteremia, with well-established standardized guidelines for fundamental procedures and key performance indicators [1]. However, ensuring the accuracy and reliability of blood cultures requires regular evaluation of adherence to these protocols in real-world clinical settings; this includes the assessment of procedural and technical aspects and timeliness and accuracy of reporting. Moreover, analyzing data, such as the distribution of isolates recovered from blood cultures, can provide insights into the quality of blood culture practices. Although the status of blood culture practices has been reported across various parameters in multiple studies, several studies, including those conducted in Korea, have identified inconsistencies between actual practices and standardized recommendations [2–6]. Such deviations can result in clinical inefficiencies and diagnostic delays [2–5,7]. Multiple performance indicators are used to evaluate the adequacy of blood culture implementation. Among these, the volume and frequency of blood collection are particularly critical for optimizing yield. In addition, proper skin disinfection techniques, appropriate use of blood culture bottles, and adequate training of healthcare personnel are essential. Following a positive culture result, timely Gram stain reporting and prompt identification and antimicrobial susceptibility testing are vital for the early diagnosis and effective management of sepsis. Therefore, ongoing assessment of current practices is necessary to identify gaps, monitor trends, and guide quality improvement efforts in blood culture procedures.

We conducted a multicenter survey of blood culture practices across university-affiliated hospitals in Korea to evaluate adherence to standardized guidelines using key performance indicators. In addition, we analyzed the distribution of blood culture isolates to identify recent trends in bacteremia and fungemia.

## Materials and methods

### Study design and setting

This is a multi-institutional, retrospective study conducted from 01/12/2024–08/03/2025 to collect statistical data on blood culture practices. A structured questionnaire was distributed to the directors of clinical microbiology laboratories at 30 university-affiliated hospitals across Korea. Completed responses, accompanied by written consent, were received from 14 institutions: Asan Medical Center, Gyeongsang National University Hospital, Chonnam National University Hospital, Chungnam National University Hospital, Dong-A University Hospital, Gyeongsang National University Changwon Hospital, Inje University Haeundae Paik Hospital, Keimyung University Dongsan Hospital, Konkuk University Medical Center, Kyung Hee University Hospital, Kyungpook National University Hospital, Pusan National University Hospital, Samsung Medical Center, and Soonchunhyang University Seoul Hospital.

### Data collection and measurement

Data collection was structured around key domains relevant to blood culture practices. The survey comprised three primary components: (1) general information of institution, including the number of hospital beds, inpatient admissions, emergency department visits, and the total number of blood cultures performed in 2023; (2) operational practices related to blood culture collection and laboratory procedures, encompassing the proportion of blood culture sets collected on the same day, the rate of repeat cultures following initial collection, the mean blood volume per culture bottle, and the proportion of bottles containing < 10 mL or < 5 mL of blood; and (3) post-analytical performance indicators, including the frequency of discordance between instrument-positive signals and subsequent culture results, the correction rate of reported results, and the proportion of Gram stain reports communicated to clinicians within 1 h or 2 h after a positive signal. The survey was designed to allow institutions to report only those variables for which data were readily available. Accordingly, only applicable responses from each institution were included in the final analysis. Except for general institutional information, blood collection volumes, and organism distribution, all data were extracted from laboratory information systems (LIS) over a 3-month period between October and December 2023.

### Data analysis

Statistical analyses of the data obtained from participating institutions were performed using MedCalc software (version 23.2.6; MedCalc Software Ltd., Ostend, Belgium).

### Ethics statement

The Institutional Review Board of Gyeongsang National University Changwon Hospital approved the study protocol (IRB-No. 2024-09-011). All participating institutions provided data with informed consent from the respective respondents.

## Results

### General characteristics

In total, 14 clinical microbiology directors from different university-affiliated hospitals participated in the survey. Of these, 8 hospitals (57.1%) had between 500 and 900 beds, whereas 6 (42.9%) had > 1,000 beds (Table 1). In 2023, the median annual number of hospitalized patients was 241,053 (interquartile range [IQR], 44,083–303,353), and the median number of annual emergency department visits was 40,158 (IQR, 29,644–46,570). The median number of blood culture tests performed annually was 43,699 (IQR, 34,065–61,617). The median number of blood culture tests per 1,000 admitted patients was 166.6 (IQR, 139.4–1,113.4).

**Table 1:** Characteristics of participating hospitals and annual blood culture numbers (N = 14)*.

General characteristics	Value
Hospital bed size, No. (%)	
500–999	8 (57.1)
≥1,000	6 (42.9)
No. of hospitalized patients per year, median (IQR)	241,053 (44,083–303,353)
No. of ED visits per year, median (IQR)	40,158 (29,644–46,570)
No. of blood cultures performed per year, median (IQR)	43,699 (34,065–61,617)
No. of blood cultures per 1,000 admitted patients, median (IQR)	166.6 (139.4–1113.4)

*Data were collected for 2023.

Abbreviations: ED, emergency department; No., number; IQR, interquartile range.

### Distribution of blood culture sets per episode

We evaluated the distribution of blood culture sets collected on the same day. Data from 12 of the 14 participating institutions were included; two institutions were excluded due to incomplete responses. The proportion of episodes with 2 sets collected on the same day was the highest, with a median of 68.9% (IQR, 55.4–86.6%), followed by 1 set at 13.0% (IQR, 6.6–30.7%) (Table 2). The proportions for 3 sets and 4 or more sets were 2.0% (IQR, 0.6–10.5%) and 1.8% (IQR, 0.3–4.1%), respectively. However, these data include pediatric patients, for whom single-set blood cultures are commonly performed in Korea. As the analysis was based on median values across institutions, the total percentage does not sum to 100%.

**Table 2:** Proportion of blood culture sets collected on the same day*.

	One set	Two sets	Three sets	Four or more sets
Median (IQR) %	13 (6.6–30.7)	68.9 (55.4–86.8)	2.0 (0.6–10.5)	1.8 (0.3–4.1)

*Based on data collected from October to December 2023 (3-month period); responses were received from 12 hospitals.

Abbreviation: IQR, interquartile range.

### Frequency of repeated culture after initial blood culture

We assessed the frequency of repeated blood cultures performed on different days (date-based). Data from 12 institutions were included in this analysis. Among patients who underwent blood culture during the study period, the median proportion who had 1 additional blood culture after the initial test was 16.7% (IQR, 11.5–18.1%),followed by 9.0% (IQR, 6.6–13.5%) for 2 additional cultures, 4.2% (IQR, 2.0–4.8%) for 3, and 2.8% (IQR, 2.1–3.3%) for 4 or more repeat cultures (Table 3).

**Table 3:** Proportion of patients undergoing additional blood cultures on different dates following the initial collection*.

Number of additional procedures	Median (IQR), %
Once	16.7 (11.5-18.1)
Twice	9.0 (6.6–13.5)
Three times	4.2 (2.0–4.8)
Four or more times	2.8 (2.1–3.3)
Proportion of repeat cultures after initial negative	29.2 (23.3–34.8)
Proportion of repeat cultures after initial positive	5.5 (3.6–6.1)

*Data collected from October to December 2023 (3-month period); responses were obtained from 12 hospitals.

Abbreviation: IQR, interquartile range.

Furthermore, among all patients who had blood cultures, the median proportion of those with a negative initial result who underwent repeat cultures (regardless of the number of repetitions) was 29.2% (IQR, 23.3–34.8%), compared to 5.5% (IQR, 3.6–6.1%) among those with a positive initial result.

### Blood culture volume per bottle

To evaluate the mean blood volume collected per culture bottle, a 2-week monitoring was conducted. At each institution (excluding 4 that did not participate in this component), 100 blood culture bottles were arbitrarily selected and manually assessed for blood volume using the scale. The median volume collected was 4.0 mL (IQR, 3.1–5.7 mL) for aerobic bottles and 4.3 mL (IQR, 3.1–6.1 mL) for anaerobic bottles (Table 4). The median proportion of aerobic bottles containing < 10 mL of blood was 99.9% (IQR, 85.0–100%), and for anaerobic bottles, 99.0% (IQR, 82.3–100%). Furthermore, the median proportion of bottles containing < 5 mL of blood was 58.0% (IQR, 34.8–74.0%) for aerobic bottles and 52.0% (IQR, 39.0–81.0%) for anaerobic bottles.

**Table 4:** Summary of blood volume metrics for aerobic and anaerobic blood culture bottles*.

	Aerobic bottle (Median [IQR])	Anaerobic bottle (Median [IQR])
Average blood volume collected (mL)	4.0 (3.1–5.7)	4.3 (3.1–6.1)
Proportion of bottles with < 10 mL (%)	99.9 (85.0–100)	99.0 (82.3–100)
Proportion of bottles with < 5 mL (%)	58.0 (34.8–74.0)	52.0 (39.0–81.0)

*Blood volumes were prospectively measured for 100 bottles over a 2-week period from 10 hospitals.

Abbreviation: IQR, interquartile range.

### Discrepancies in the test results and post-test reporting practices

Data from 10 institutions were analyzed to evaluate the frequency of discrepancies between instrument-detected positive signals and the recovery of identifiable organisms. The median proportion of such false-positive cases was 0.8% (IQR, 0.36–1.85%). The correction rate of blood culture results following initial reporting was assessed based on data from 13 institutions, with a median of 0.01% (IQR, 0–0.03%).

Among the 9 institutions that reported data on Gram stain turnaround times, the median proportion of results communicated within 1 h of a positive signal was 7.1% (IQR, 3.8–40.5%), whereas 16.7% (IQR, 9.3–50.7%) were reported within 2 h.

## Discussion

Blood cultures are a cornerstone in the diagnosis and management of sepsis, as positive results directly inform antimicrobial selection and treatment duration, thereby significantly influencing patient outcomes. The blood culture positivity rate is a key performance indicator and may vary depending on patient characteristics, including disease severity, age, comorbidities, and immune status. A recent national survey in Korea reported a median positivity rate of 7.9% (IQR, 6.58–9.93%), emphasizing the variability across institutions [6]. Multiple factors contribute to this variation, including institutional type and size, patient population characteristics, and clinical decision-making processes. From a laboratory standpoint, strict adherence to standardized protocols and consistent monitoring of performance indicators are essential for maintaining high-quality diagnostic practices [8,9]. Accordingly, regular evaluation of blood culture implementation in clinical settings is critical to ensure optimal outcomes. We collected and analyzed statistical data from university-affiliated hospitals nationwide to assess blood culture performance and identify areas requiring improvement.

In adults, it is generally recommended that at least two sets of blood cultures be obtained within a 24-h period to improve diagnostic yield and help distinguish true bacteremia from contamination [1]. In this survey, two-set blood cultures accounted for the largest proportion (68.9%).; however, single-set collections were still reported in 13.0% of cases. Although the U.S. Centers for Disease Control and Prevention do not specify a defined threshold for acceptable single-set culture rates, it recommends routine monitoring and institutional reporting of this indicator [10]. Furthermore, in instances where only a single set is received, clinicians should be informed about the potential for false-negative results, and additional culture sets should be recommended as clinically appropriate [11]. Routine tracking of single-set usage and dissemination of findings to relevant clinical teams may aid in identifying gaps in practice and inform targeted educational or process improvement initiatives. Previous studies have demonstrated substantially higher positivity rates with two sets (18%) compared to one set (9%) in adults, and similar trends have been observed in pediatric populations, with positivity rates of 8.9% for two sets versus 1.0% for a single set [12,13] These findings emphasize the importance of age-appropriate blood culture practices across patient groups. A limitation of our study is the potential inclusion of pediatric cases, for whom single-set cultures are standard practice in Korea. Moreover, the analysis was based on prescriptions issued on the same day, which may not strictly align with a 24-h collection window. Notably, there are no age-specific guidelines for the pediatric population, and single-set collection remains the norm in this group.

Although repeat blood cultures following an initial set may enhance diagnostic yield in select clinical scenarios—such as infective endocarditis, persistent *S. aureus* bacteremia, and candidemia, where results inform antimicrobial adjustments or determine treatment duration—they may contribute to unnecessary antimicrobial use, overdiagnosis, and resource overutilization when performed indiscriminately [14]. In this survey, additional blood cultures were ordered in 16.7% and 9.0% of cases for one and two additional sets, respectively. As this analysis was based on calendar dates rather than a strict 24-h timeframe, this methodology may have influenced the reported proportions. Regardless, appropriate use of repeat blood cultures should be closely monitored, and active communication with attending physicians is essential to ensure clinical justification. Notably, repeat cultures were more commonly ordered after an initially negative result (29.2%) compared to an initially positive result (5.5%). These findings are consistent with those reported by Tabriz et al., who found that 31.6% of repeat cultures were ordered regardless of initial results, primarily due to persistent fever [15]. In that study, 83.4% of repeat cultures remained negative, 9.1% yielded the same organism, and 2.5% identified new pathogens. Current guidelines for repeat blood cultures are limited and largely context-dependent. For example, repeat cultures are recommended to confirm microbial clearance in patients with central line-associated bloodstream infections [16]. Similarly, repeat cultures are advised in cases of infective endocarditis, methicillin-resistant *S. aureus* (MRSA) bacteremia, and candidemia when the initial culture is positive, to confirm pathogen clearance and guide therapy duration [17]. Repeat blood cultures may be appropriate in specific clinical scenarios, such as suspected treatment failure, infections caused by multidrug-resistant organisms, prolonged neutropenia, unexplained or relapsing sepsis, clinical deterioration, or suspected new-onset infections [14]. Conversely, in cases of *Streptococcus* or *Enterobacterales* bacteremia where appropriate antimicrobial therapy has been initiated and the source of infection is controlled, repeat blood cultures are generally not recommended [18]. Rather than routinely obtaining repeat cultures in response to persistent fever, emphasis should be placed on the interpretation of initial culture results—ideally obtained before the initiation of antimicrobial therapy. Therefore, the decision to perform repeat blood cultures should be made judiciously, guided by clinical context and supported by evidence. Furthermore, strict adherence to fundamental blood culture techniques—such as proper skin disinfection and adequate blood volume collection—remains essential in minimizing false-negative results and reducing the need for unnecessary repeat testing. However, because the specific clinical indications for repeat blood cultures were not available in this survey, the findings should be interpreted as descriptive rather than as a performance indicator.

Current guidelines recommend collecting 20–30 mL of blood per set, typically 10 mL per bottle [1]. However, in this survey, more than 99% of both aerobic and anaerobic bottles contained < 10 mL, with 58% and 52%, respectively, containing < 5 mL. The measured mean volume per bottle ranged from 4.0–4.3 mL, significantly below the recommended threshold. These findings emphasize a substantial discrepancy between guideline-based recommendations and actual clinical practice [19]. Given that blood volume is one of the most critical determinants of blood culture sensitivity, targeted interventions are essential to bridge this gap. Potential strategies include enhanced education and training programs for phlebotomists or medical personnel, increased awareness of the importance of adequate sample volume, routine feedback on blood volume via automated culture monitoring systems, and institution-specific assessments to identify and address barriers to optimal blood collection [20,21].

Following a positive signal from an automated blood culture system, prompt Gram staining and rapid communication with clinicians are critical for the effective management of sepsis. In this survey, Gram staining was performed within 1 h of the positive signal in only 7.1% of cases, and within 2 h in 16.7%, indicating that real-time reporting is frequently hindered by practical workflow constraints in clinical settings. Schifman et al. [22] revealed that the median turnaround time (TAT) from organism growth detection to Gram stain result reporting is approximately 45 min, with the Gram staining process itself accounting for a median of 25 min—the most time-consuming step. These discrepancies may reflect differences in laboratory operating hours, staffing levels, recognition of the clinical importance of critical value reporting, and overall workflow organization. To improve TAT from detection to Gram stain reporting, minimizing delays between signal detection and staining is essential, ideally by implementing near-real-time processes. If feasible, operating a 24-hour microbiology laboratory, conducting regular analyses of performance indicators to identify workflow delays and reorganize staff assignments accordingly, and actively utilizing automated alert systems for positive blood culture signals can all serve as effective strategies to enhance the timeliness and efficiency of laboratory processes. Where feasible, the incorporation of rapid diagnostic methods using positive blood culture broth—such as direct matrix-assisted laser desorption/ionization time-of-flight mass spectrometry or the FilmArray BCID panel (BioFire, Salt Lake City, UT)—should also be considered to enhance pathogen identification and support timely antimicrobial intervention [23,24].

Our study has several limitations. First, it was conducted retrospectively in only 14 university-affiliated hospitals, which may limit the generalizability of the findings to the broader national context, particularly to small- or medium-sized institutions. However, in depth data analysis revealed the current status of key indicators and area of improvement of blood cultures in those university-affiliated hospitals where the clinical severity may be highest. Second, the inclusion of pediatric blood culture data may have influenced findings related to the number of sets collected. Third, the data also did not differentiate between samples drawn from peripheral veins and those obtained through central venous catheters, which may have influenced the analysis of the appropriateness of the number of blood culture sets collected. Finally, the relatively short, 3-month data collection period and the fact that certain variables—such as the number of sets collected on the same day and the frequency of repeat cultures—were available from only a subset of hospitals may have introduced additional bias. We set the period of collection of data for 3 months, because the whole data would be too much for 1 year. Several data analyses, such as repeat culture were performed manually from the data received from LIS. In addition, the number of culture bottles assessed for blood volume in each institution was too small to draw relevant conclusions. However, the total number of bottles reached to 2,000 to analyze the blood volume.

## Conclusions

This nationwide analysis of blood culture practices in university-affiliated hospitals provides valuable insights into the current state of implementation and performance of quality indicators in Korea. Although certain aspects—such as test ordering appropriateness—are being adequately addressed, significant discrepancies persist between recommended guidelines and actual practices in areas such as blood volume adequacy, indications for repeat cultures, and the timeliness of Gram stain reporting. Our findings emphasize the need for ongoing evaluation of institutional blood culture practices to drive quality improvement throughout the diagnostic process. Therefore, targeted interventions should be developed to address identified deficiencies, and efforts to adhere the standard guidelines of blood cultures must be further reinforced.

## References

[1] Clinical and Laboratory Standards Institute. Principles and procedures for blood cultures. M47. Wayne, PA: CLSI; 2022.

[2] Åkerlund A, Petropoulos A, Malmros K, Tängdén T, Giske CG. Blood culture diagnostics: a Nordic multicentre survey comparison of practices in clinical microbiology laboratories. Clin Microbiol Infect. 2022;28(5):731.e1-731.e7. doi: 10.1016/j.cmi.2021.09.003 34537364

[3] Temkin E, Biran D, Braun T, Schwartz D, Carmeli Y. Analysis of blood culture collection and laboratory processing practices in Israel. JAMA Netw Open. 2022;5(10):e2238309. doi: 10.1001/jamanetworkopen.2022.38309 36282502 PMC9597385

[4] Shin JH, Song SA, Kim M, Kim S. Nationwide survey of blood culture performance regarding skin disinfection, blood collection and laboratory procedures. Korean J Clin Microbiol. 2011;14(3):91. doi: 10.5145/kjcm.2011.14.3.91

[5] Kim YA, Kim D, Yong D, Lee K. Nationwide survey of blood culture protocol in clinical microbiology laboratories in Korea. Ann Clin Microbiol. 2016;19(4):97. doi: 10.5145/acm.2016.19.4.97

[6] Song SA, Park S, Woo K, Kim YK, Kim J, Kim S. A nationwide survey on standard blood culture protocols and quality improvement for the diagnosis of sepsis. Lab Med Online. 2025;15(3):239–46. doi: 10.47429/lmo.2025.15.3.239

[7] Elvy J, Addidle M, Andersson H-S, Black V, Drinković D, Howard J, et al. A national audit of performance standards for blood cultures in Aotearoa New Zealand: opportunities for improvement. N Z Med J. 2022;136(1568):65–71. doi: 10.26635/6965.5930 36657076

[8] He M, Huang S, Xiong J, Xiao Q. Improving adherence to facility protocol and reducing blood culture contamination in an intensive care unit: a quality improvement project. Aust Crit Care. 2020;33(6):546–52. doi: 10.1016/j.aucc.2020.03.002 32417183

[9] Palavecino EL, Campodónico VL, She RC. Laboratory approaches to determining blood culture contamination rates: an ASM laboratory practices subcommittee report. J Clin Microbiol. 2024;62(2):e0102823. doi: 10.1128/jcm.01028-23 38051070 PMC10865823

[10] Centers for Disease Control and Prevention CDC. Sub-measure: single-set blood culture rate. Centers for Disease Control and Prevention. 2024. Accessed 2025 July 10. https://www.cdc.gov/lab-quality/php/prevent-adult-blood-culture-contamination/sub-measure-single-set.html

[11] Centers for Disease Control and Prevention CDC. Prevent adult blood culture contamination: a quality tool. CDC. 2023. Accessed 2025 July 10. https://www.cdc.gov/hai/bloodstream/bcc-quality-tool.html

[12] Patel R, Patel N, Patel R. Assessment of factors influencing the positivity of blood culture by BacT/ALERT®3D microbial detection system: a cross-sectional observational study. J Clin Diag Res. 2022. doi: 10.7860/jcdr/2022/56676.16759

[13] Zalmanovich A, Temkin E, Biran D, Carmeli Y. The yield of one vs. two blood cultures in children: under-detection and over-testing. Antibiotics (Basel). 2024;13(2):113. doi: 10.3390/antibiotics13020113 38391499 PMC10886363

[14] Mushtaq A, Bredell BX, Soubani AO. Repeating blood cultures after initial bacteremia: when and how often?. Cleve Clin J Med. 2019;86(2):89–92. doi: 10.3949/ccjm.86a.18001 30742578

[15] Tabriz MS, Riederer K, Baran J Jr, Khatib R. Repeating blood cultures during hospital stay: practice pattern at a teaching hospital and a proposal for guidelines. Clin Microbiol Infect. 2004;10(7):624–7. doi: 10.1111/j.1469-0691.2004.00893.x 15214874

[16] Baang JH IK, Nagel J. Diagnosis and Treatment of Catheter-Related Bloodstream Infection. Michigan Medicine University of Michigan; 2023. 37014953

[17] Liu C, Bayer A, Cosgrove SE, Daum RS, Fridkin SK, Gorwitz RJ, et al. Clinical practice guidelines by the infectious diseases society of america for the treatment of methicillin-resistant Staphylococcus aureus infections in adults and children. Clin Infect Dis. 2011;52(3):e18-55. doi: 10.1093/cid/ciq146 21208910

[18] Fabre V, Sharara SL, Salinas AB, Carroll KC, Desai S, Cosgrove SE. Does this patient need blood cultures? A scoping review of indications for blood cultures in adult nonneutropenic inpatients. Clin Infect Dis. 2020;71(5):1339–47. doi: 10.1093/cid/ciaa039 31942949

[19] Shin JH, Song SA, Kim M-N, Lee NY, Kim E-C, Kim S, et al. Comprehensive analysis of blood culture performed at nine university hospitals in Korea. Korean J Lab Med. 2011;31(2):101–6. doi: 10.3343/kjlm.2011.31.2.101 21474985 PMC3115996

[20] Lee S, Kim SC, Kim S. Educational intervention to improve blood culture indicators in a secondary-care hospital. Ann Clin Microbiol. 2021;24(1):1–9. doi: 10.5145/acm.2021.24.1.1

[21] Chen Y, Dai Y, Zhou Y, Huang Y, Jin Y, Geng Y, et al. Improving blood culture quality with a medical staff educational program: a prospective cohort study. Infect Drug Resist. 2023;16:3607–17. doi: 10.2147/IDR.S412348 37309379 PMC10257920

[22] Schifman RB, Meier FA, Souers RJ. Timeliness and accuracy of reporting preliminary blood culture results: a College of American Pathologists Q-probes study of 65 institutions. Arch Pathol Lab Med. 2015;139(5):621–6. doi: 10.5858/arpa.2014-0258-CP 25927146

[23] Bianco G, Comini S, Boattini M, Ricciardelli G, Guarrasi L, Cavallo R. MALDI-TOF MS-based approaches for direct identification of gram-negative bacteria and Bla(KPC)-carrying plasmid detection from blood cultures: a three-year single-centre study and proposal of a diagnostic algorithm. Microorganisms. 2022;11(1). 10.3390/microorganisms11010091 PMC9860562 36677383

[24] Yang M, Tao C. Diagnostic efficiency of the FilmArray blood culture identification (BCID) panel: a systematic review and meta-analysis. J Med Microbiol. 2023;72(9):10.1099/jmm.0.001608. doi: 10.1099/jmm.0.001608 37712641

